# Mesenchymal Tumor of the Hand Causing Tumor-Induced Osteomalacia

**DOI:** 10.7759/cureus.104071

**Published:** 2026-02-22

**Authors:** Andrew Cecil, Meti Shagi, Syed Basit Haider, Syed Ali A Gardezi

**Affiliations:** 1 Internal Medicine, Aurora Health Care, Milwaukee, USA; 2 Endocrinology, Diabetes and Metabolism, Aurora Health Care, Milwaukee, USA

**Keywords:** fgf23, hand tumor, hypophosphatemia, phosphaturic mesenchymal tumor, tumor-induced osteomalacia

## Abstract

Tumor-induced osteomalacia (TIO) is an ultra-rare paraneoplastic syndrome caused by excessive secretion of fibroblast growth factor 23 (FGF23) from phosphaturic mesenchymal tumors, leading to renal phosphate wasting, hypophosphatemia, and osteomalacia. Diagnosis is frequently delayed due to nonspecific symptoms and the difficulty of localizing small, often indolent tumors. We report a 55-year-old male patient who presented with a 16-month history of progressive muscle weakness, chronic bone pain, and multiple bilateral metatarsal fractures. Laboratory evaluation demonstrated profound hypophosphatemia (1.6 mg/dL), markedly elevated FGF23 levels (455 RU/mL), and renal phosphate wasting (tubular reabsorption of phosphate at 35%, tubular maximum phosphate reabsorption per glomerular filtration rate (TmP/GFR) at 1.04 mg/dL). Initial fluorodeoxyglucose positron emission tomography/computed tomography (FDG-PET/CT) was non-localizing. Following referral to a tertiary center, specialized re-interpretation of imaging identified faint focal uptake in the right third metacarpal. This finding was confirmed with technetium-99m sestamibi scintigraphy and magnetic resonance imaging, which revealed a 1.9 × 1.8 × 1.2 cm soft-tissue mass within the palmar tissues. Surgical excision was performed, and histopathology confirmed a phosphaturic mesenchymal tumor with positive FGF23 in situ hybridization. Postoperatively, serum phosphorus levels normalized, confirming biochemical cure. This case underscores the diagnostic challenges associated with TIO, particularly tumor localization, and highlights the critical role of expert imaging interpretation. Complete surgical resection remains the definitive treatment, resulting in rapid biochemical normalization and clinical improvement. The unusual hand location and initially non-localizing imaging emphasize the importance of a systematic, multidisciplinary approach in the evaluation and management of this rare but highly treatable condition.

## Introduction

Tumor-induced osteomalacia (TIO) is an ultra-rare paraneoplastic disorder caused by excessive secretion of fibroblast growth factor 23 (FGF23), usually from small, slow-growing mesenchymal tumors, leading to hypophosphatemia and defective bone mineralization [1,2]. Patients often present with nonspecific symptoms such as bone pain, muscle weakness, and fractures, resulting in frequent misdiagnosis and prolonged delays to diagnosis [3].

FGF23 disrupts phosphate homeostasis by reducing renal phosphate reabsorption and suppressing conversion of 25-hydroxyvitamin D to its active form, 1,25-dihydroxyvitamin D. This leads to persistent hypophosphatemia, low or inappropriately normal calcitriol, and impaired bone mineralization [1,2]. Laboratory findings typically include low serum phosphate, elevated or inappropriately normal FGF23, low-to-normal 1,25-dihydroxyvitamin D, and elevated alkaline phosphatase [3].

Phosphaturic mesenchymal tumors are usually small (median 2.7-3.0 cm) and can arise in soft tissue or bone, with the lower extremities and pelvis being most common [4]. Due to this, tumor localization is challenging and requires a systematic approach, beginning with functional imaging, preferably somatostatin receptor positron emission tomography/computed tomography (PET/CT), which offers higher sensitivity than fluorodeoxyglucose-PET (FDG-PET) or octreotide scans [5,6]. Once identified, surgical excision with wide margins is curative in most cases [4]. For patients with unresectable or non-localized tumors, medical therapy with phosphate, active vitamin D, or the anti-FGF23 antibody burosumab provides successful disease management [7-9].

## Case presentation

A 55-year-old male patient with a history of hypertension, hyperlipidemia, reactive airway disease, and mitral valve prolapse status post mitral valve repair, presented to the endocrinology clinic with multiple recent bilateral metatarsal fractures and 16 months of progressive muscle weakness. His ambulation was significantly limited, necessitating cessation of work as an electrician due to pain.

The patient reported persistent sternal pain since undergoing open-heart surgery in 2013, as well as chronic metatarsal bone pain. Additional pain involved the hips, knees, and ribs. He had a history of military deployment to Afghanistan, during which he remained physically active without notable trauma or fractures. He also noted an increase in shoe size from 11.5 to 13. There was no significant family history of endocrine disorders or fractures.

**Figure 1:**
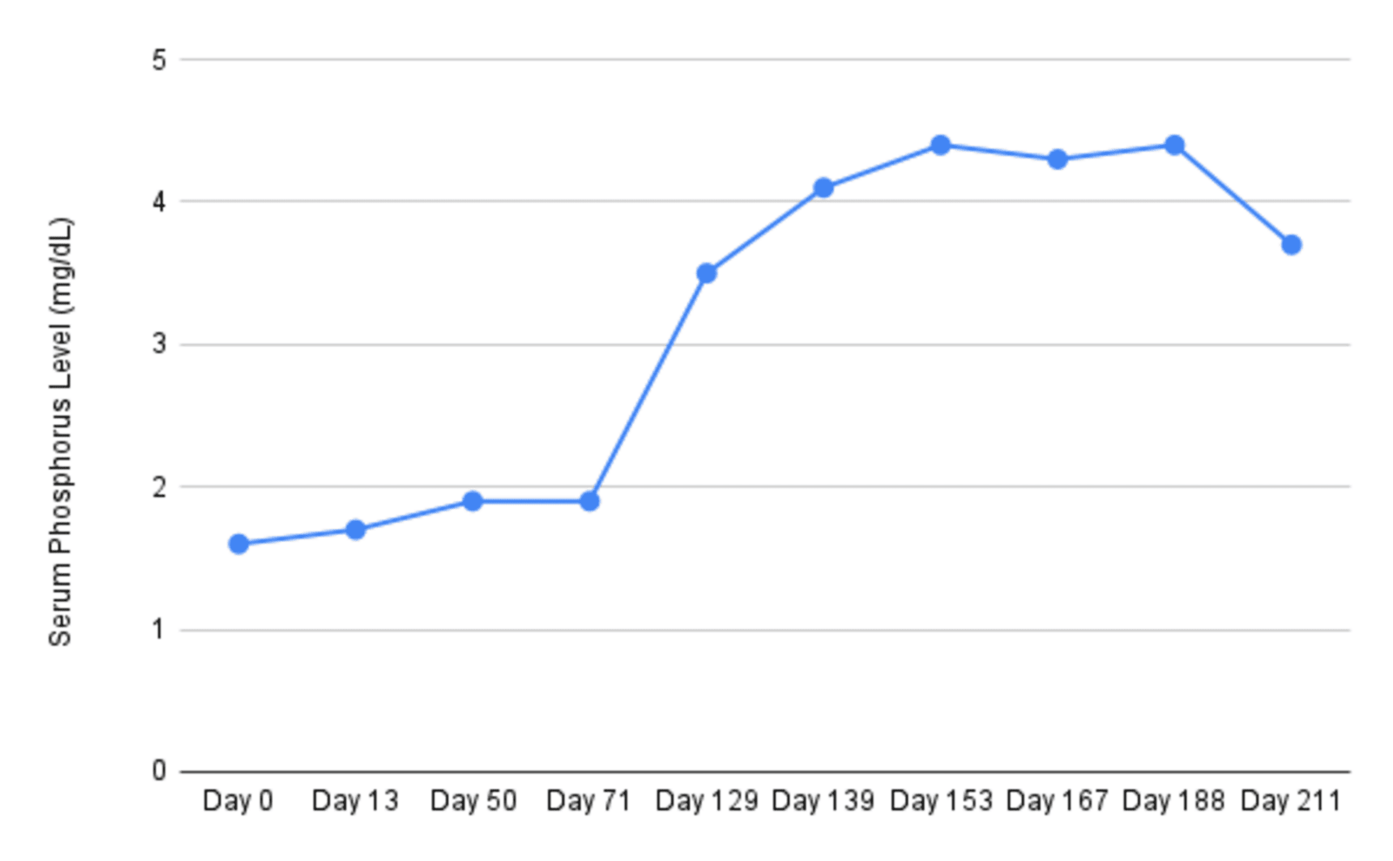
Serum phosphate level over disease course and after tumor resection (9/15/2014)

**Figure 2:**
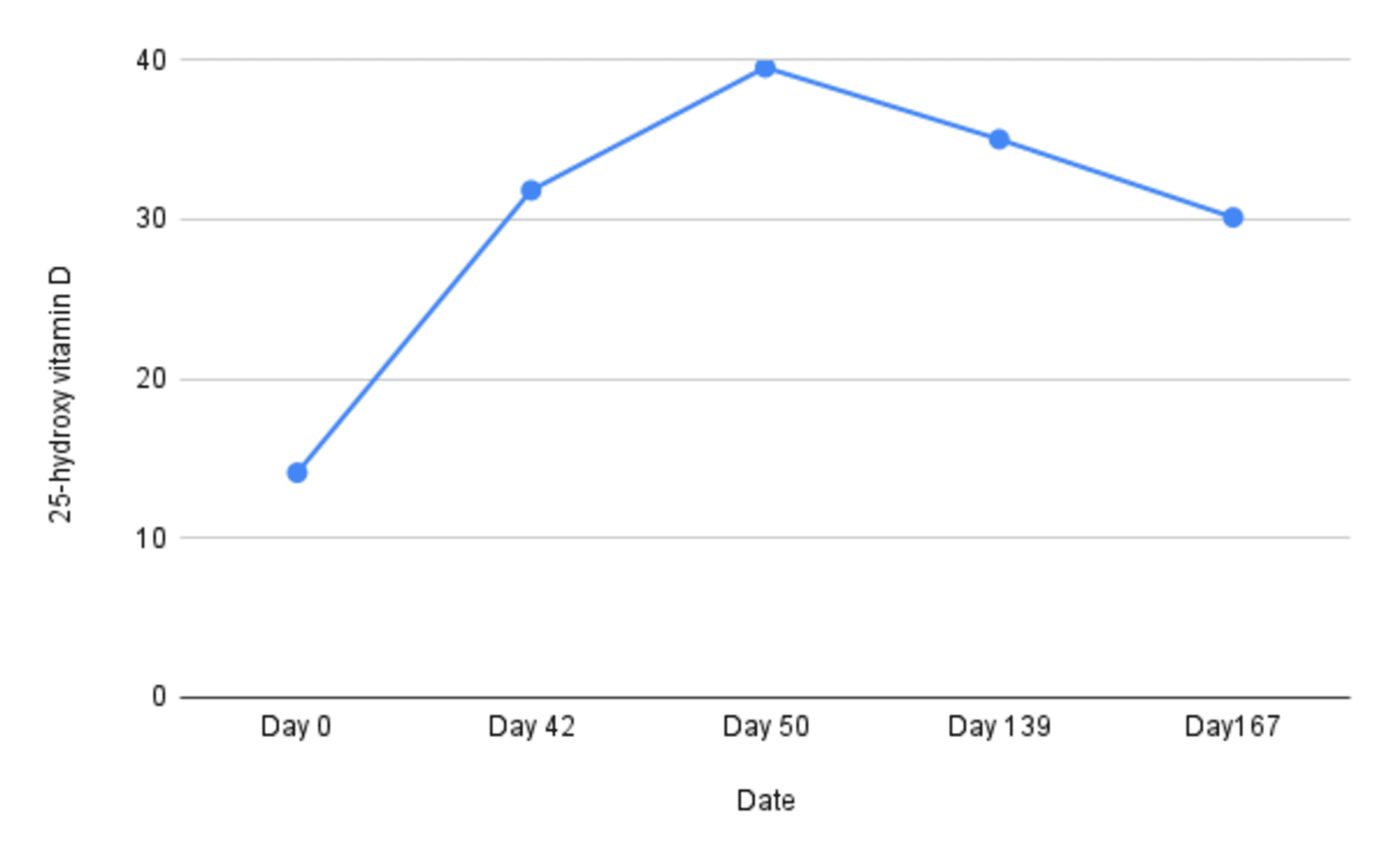
Serum 25-hydroxy vitamin D levels

On examination, he demonstrated a markedly slow gait and required a cane for ambulation. Vital signs were stable: blood pressure 106/78 mmHg, heart rate 84 bpm, respiratory rate 18/min. Laboratory evaluation revealed hypophosphatemia (serum phosphate 1.6 mg/dL; reference range: 2.4-4.7 mg/dL), elevated alkaline phosphatase (161 U/L; reference range: 45-117 U/L), and 25-OH vitamin D (14.1 ng/mL; reference range: 30-100 ng/mL). His 24-hour urine phosphate was 1.4 g (reference range: 0.4-1.3 g/24 h), tubular reabsorption of phosphate (TRP) was 35% (reference range: 85-95%), and tubular maximum phosphate corrected for glomerular filtration rate (TmP/GFR) was 1.04 mg/dL (reference range: 2.8-4.4 mg/dL), consistent with renal phosphate wasting (Figures 1, 2).

FGF23 was markedly elevated at 455 RU/mL (Table 1).

**Table 1:** Initial laboratory results with corresponding reference ranges TmP/GFR: tubular maximum phosphate reabsorption per glomerular filtration rate.

Laboratory tests	Result	Reference range
Serum phosphorus (mg/dL)	1.6	2.4–4.7
Alkaline phosphatase (U/L)	161	45–117
24-hour urine phosphorus (g/24hr)	1.4	0.4-1.3
TmP/GFR (mg/dL)	1.04	2.8-4.4
Vitamin D (ng/mL)	14.1	30-100
Parathyroid hormone (pg/mL)	66	14-72
Fibroblast growth factor 23 (RU/mL)	455	44-215

Other laboratory studies, including serum and urine protein electrophoresis, testosterone, insulin-like growth factor-1, and intact parathyroid hormone, were within normal limits. The patient was initiated on vitamin D supplementation (4000 units daily) and oral potassium phosphate therapy (1 gram three times daily).

**Figure 3:**
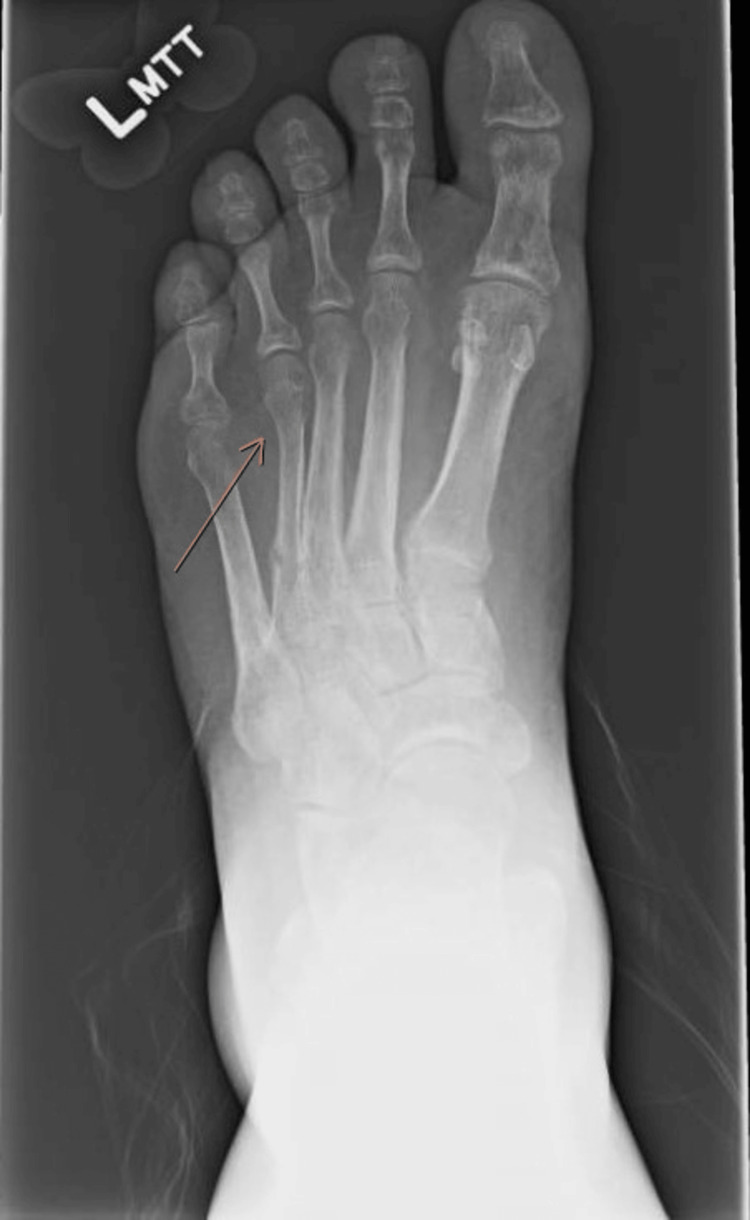
Radiograph of the left foot Demonstrates healing of a proximal diaphyseal fracture of the fourth metatarsal with interval healing, along with inferior and posterior calcaneal spurring, and generalized osteopenia.

**Figure 4:**
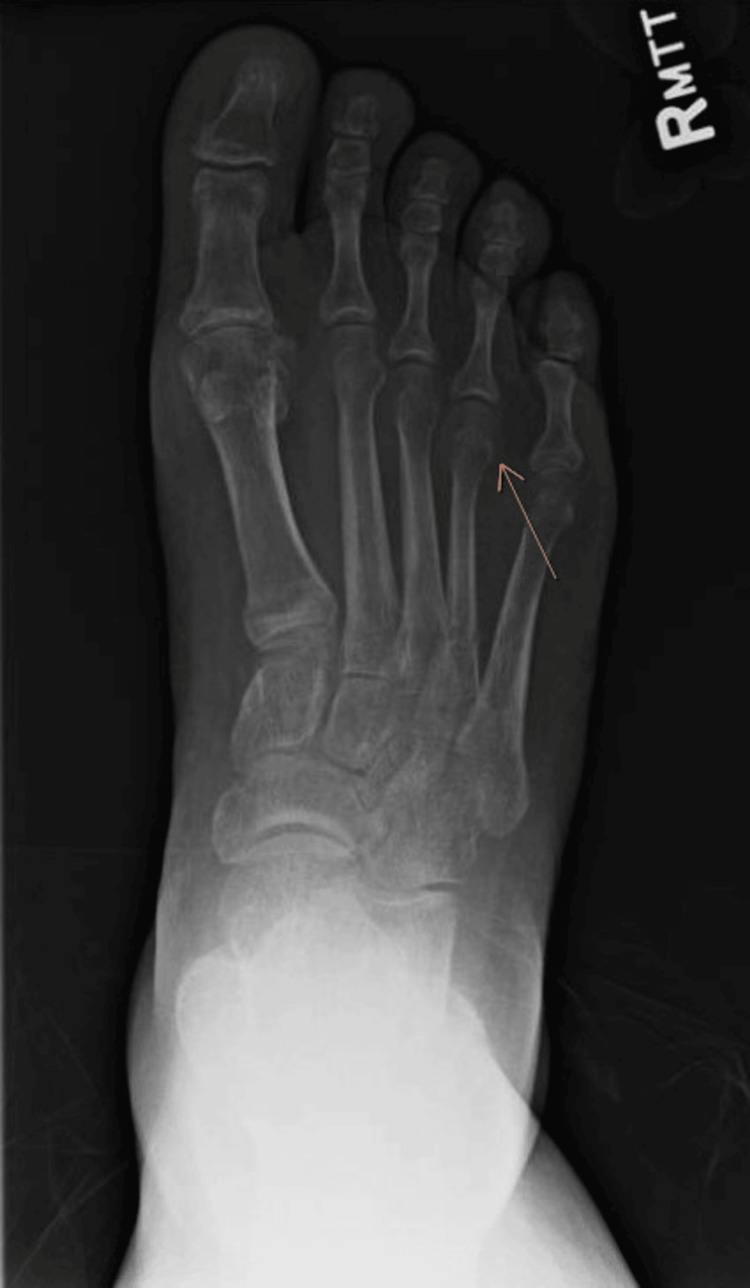
Radiograph of the right foot Demonstrates healing proximal diaphyseal fracture at the fourth metatarsal and presence of inferior calcaneal spurring with generalized osteopenia.

Radiographs of the feet showed healing bilateral fourth metatarsal fractures with diffuse osteopenia (Figures 3, 4).

**Figure 5:**
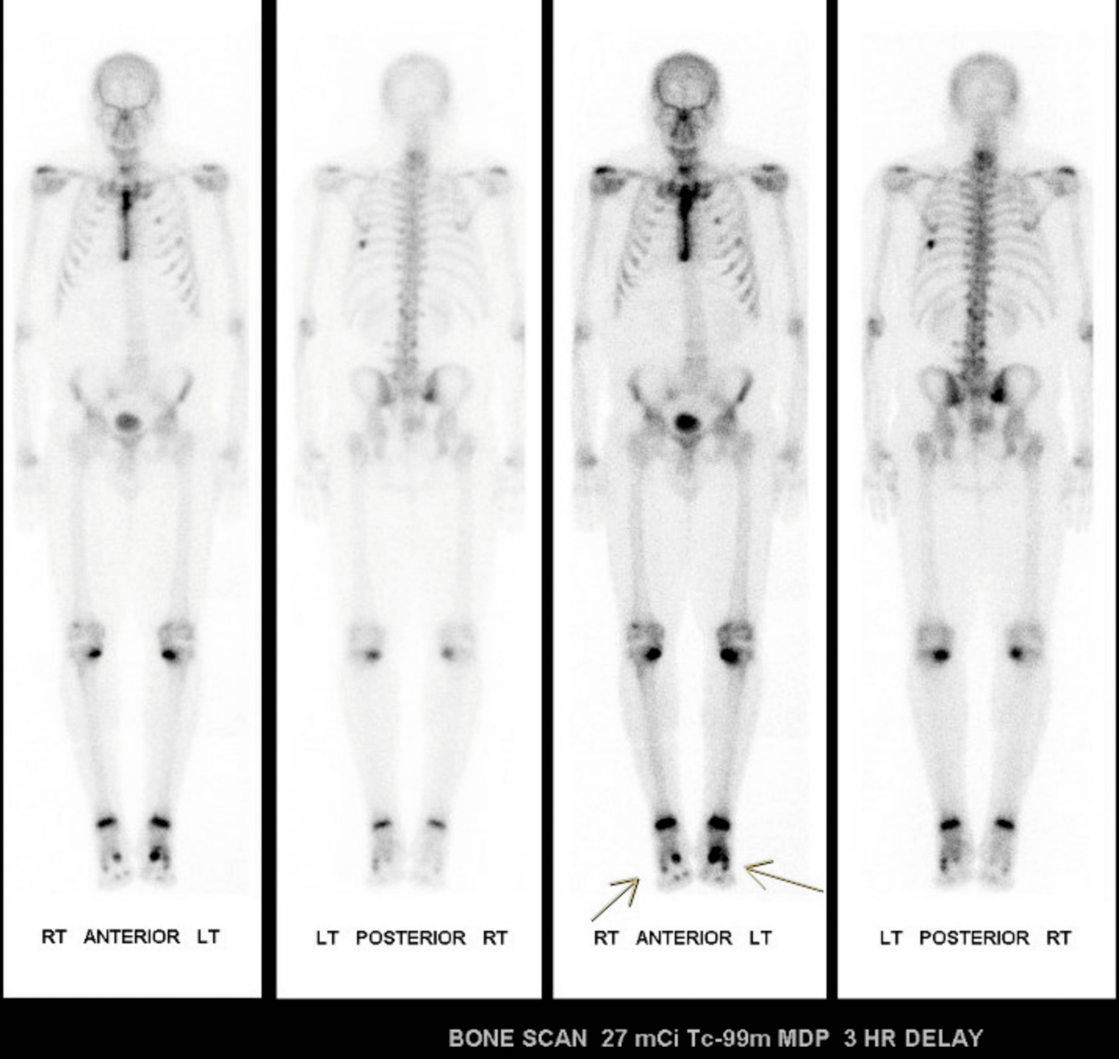
Bone scan Demonstrates increased uptake in the left first metatarsal, distal tibias bilateral, bilateral medial tibias and multiple less intense radiotracer uptake within the feet. Increased uptake in the sternum and ribs is consistent with recent open heart surgery.

Bone scan revealed mild increased uptake at the base of the first metatarsal, moderate uptake in the distal tibias, and marked uptake in the sternum, left ribs, and feet (Figure 5).

**Figure 6:**
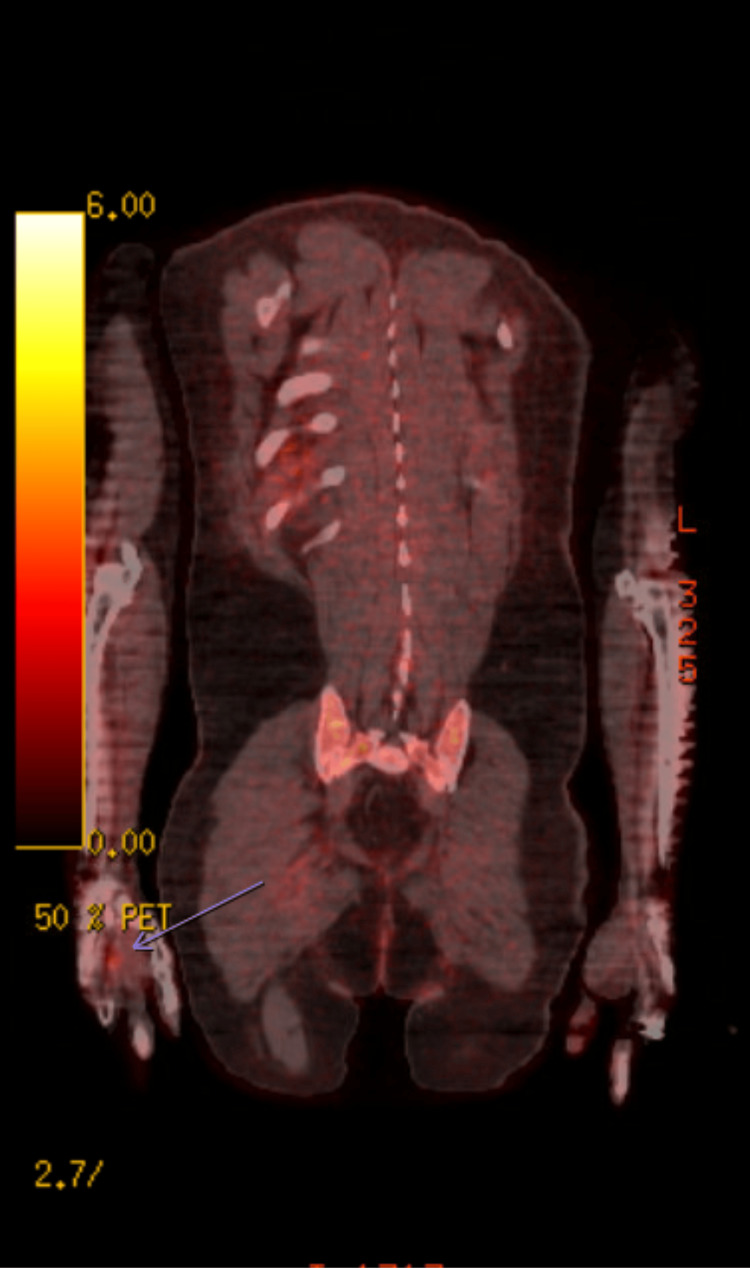
Faint focal FDG activity within the right third metacarpal visualized on the reinterpretation of PET/CT FDG: fluorodeoxyglucose; PET/CT: positron emission tomography/computed tomography.

Initial PET/CT did not identify abnormal FDG uptake concerning for malignancy and was considered non-localizing. A re-interpretation of imaging identified faint focal FDG uptake in the right third metacarpal (Figure 6).

**Figure 7:**
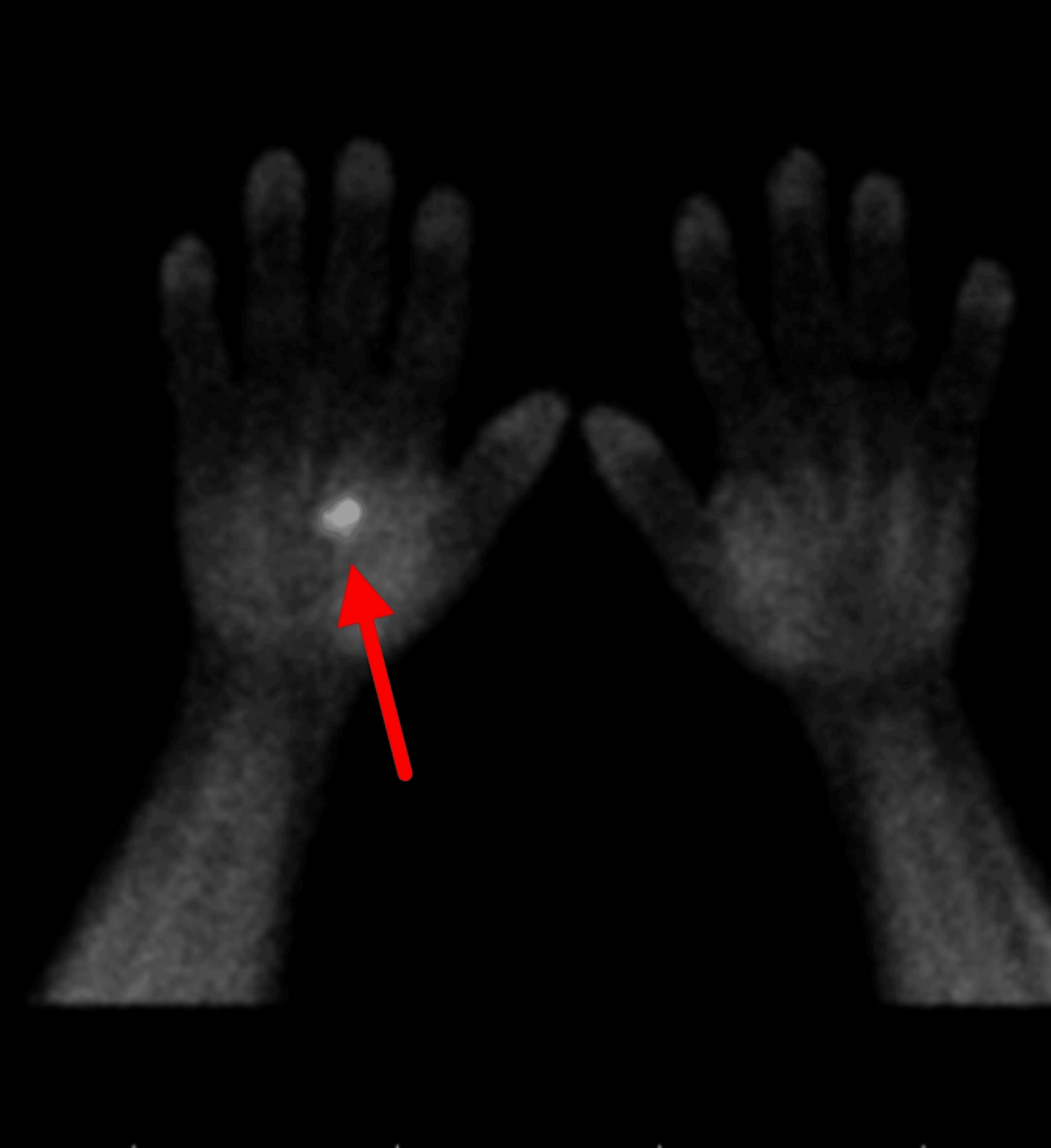
Increased radiotracer uptake noted in the right palm on the sestamibi scan

Technetium-99m sestamibi scanning confirmed tracer uptake in the right palm (Figure 7).

**Figure 8:**
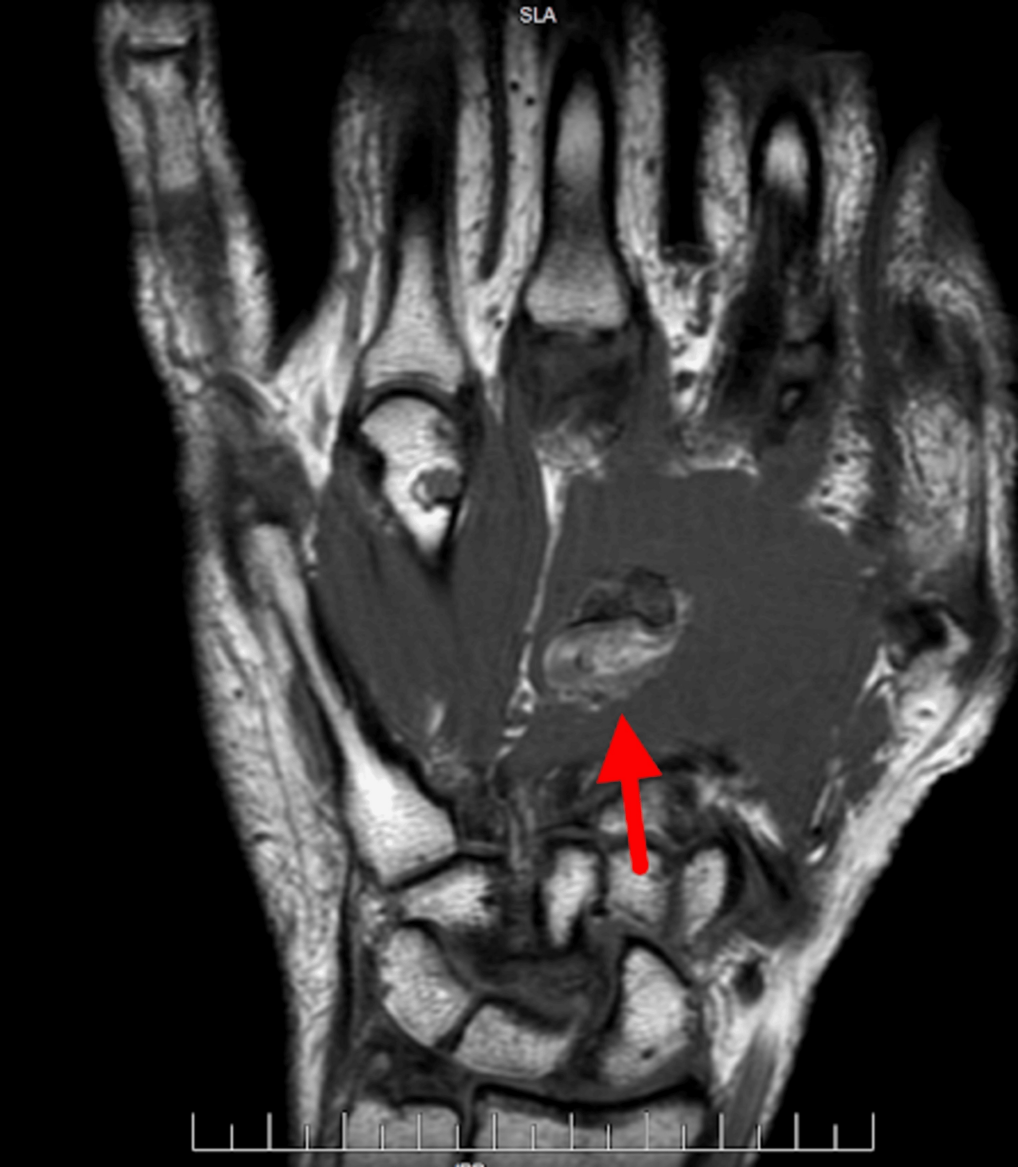
MRI imaging Demonstrates a 1.9 × 1.8 × 1.2 cm heterogeneously enhancing ovoid soft-tissue mass within the palmar tissues, centered over the proximal shaft of the third metacarpal and extending into the interspace between the second and the third metacarpals.

MRI demonstrated a 1.9 × 1.8 × 1.2 cm heterogeneously enhancing ovoid soft-tissue mass within the palmar tissues, centered over the proximal shaft of the third metacarpal and extending into the interspace between the second and third metacarpals, consistent with a phosphaturic mesenchymal tumor (Figure 8).

The patient underwent surgical excision of the mass. Histopathology confirmed a phosphaturic mesenchymal tumor with positive FGF23 mRNA on in situ hybridization. Postoperatively, serum phosphorus normalized within two months of the procedure.

## Discussion

This case emphasizes the diagnostic challenges of TIO, including its nonspecific symptoms and the difficulty in localizing the tumor. Notably, the patient experienced 16 months of progressive weakness, fractures, and chronic bone pain. These symptoms are consistent with the insidious course reported in the literature, where diagnosis can be delayed over several years [3].

Laboratory findings, including marked hypophosphatemia, elevated FGF23, and renal phosphate wasting, were diagnostic in this context. These values are consistent with reported series, confirming that biochemical evaluation is critical for identifying TIO [1,3].

Tumor localization proved challenging in this patient, as initial FDG-PET/CT was non-diagnostic. However, a specialized review ultimately demonstrated faint uptake in the right third metacarpal, which was confirmed by sestamibi scintigraphy and MRI. This sequence underscores the critical role of expert interpretation and stepwise functional imaging, particularly using somatostatin receptor-based PET/CT, for accurate localization and surgical planning [5,6]. Of note, this case predates the 2023 Global Guidance for TIO, which now recommends somatostatin receptor-based PET/CT (e.g., 68Ga-DOTATATE) as first-line functional imaging due to its superior sensitivity over FDG-PET/CT [2,10,11]. 

Histopathology confirmed a phosphaturic mesenchymal tumor with positive FGF23 in situ hybridization, a highly sensitive and specific diagnostic method [12]. Postoperative normalization of serum phosphate demonstrated cure, consistent with the rapid biochemical response expected following complete excision [13-17].

Key clinical lessons from this case include the need to consider TIO in any patient with unexplained hypophosphatemia, bone pain, and fractures, and the utility of early, systematic imaging to localize the tumor. Complete surgical resection remains essential to prevent recurrence, while patients with unresectable or non-localized disease benefit from phosphate, vitamin D, or burosumab therapy. This case highlights that multidisciplinary care optimizes outcomes in this rare but treatable condition [1,3,5,6,8,10,18].

## Conclusions

This case highlights the diagnostic and therapeutic challenges of TIO and demonstrates the excellent outcomes achievable with complete surgical resection. Prolonged nonspecific musculoskeletal symptoms in the setting of hypophosphatemia and elevated FGF23 levels should raise clinical suspicion for TIO. Accurate tumor localization is critical and often requires advanced functional imaging interpreted at specialized centers.

Surgical excision with wide margins remains the definitive treatment, typically resulting in rapid normalization of serum phosphorus and symptomatic improvement. In patients with unresectable or non-localizing tumors, medical therapy with phosphate, active vitamin D, or burosumab offers effective disease control by targeting FGF23 excess. Early recognition, systematic diagnostic evaluation, and multidisciplinary management are essential to optimize outcomes in this rare but treatable condition. 
